# A Comparative Study of 5S rDNA Non-Transcribed Spacers in Elaeagnaceae Species

**DOI:** 10.3390/plants10010004

**Published:** 2020-12-23

**Authors:** Oleg S. Alexandrov, Olga V. Razumova, Gennady I. Karlov

**Affiliations:** All-Russia Research Institute of Agricultural Biotechnology, Timiryazevskaya 42, 127550 Moscow, Russia; razumovao@gmail.com (O.V.R.); karlovg@gmail.com (G.I.K.)

**Keywords:** Elaeagnaceae, *Elaeagnus*, *Shepherdia*, 5S rDNA, non-transcribed spacers, polymorphism, PCR, molecular evolution

## Abstract

5S rDNA is organized as a cluster of tandemly repeated monomers that consist of the conservative 120 bp coding part and non-transcribed spacers (NTSs) with different lengths and sequences among different species. The polymorphism in the 5S rDNA NTSs of closely related species is interesting for phylogenetic and evolutional investigations, as well as for the development of molecular markers. In this study, the 5S rDNA NTSs were amplified with universal 5S1/5S2 primers in some species of the Elaeagnaceae Adans. family. The polymerase chain reaction (PCR) products of five *Elaeagnus* species had similar lengths near 310 bp and were different from *Shepherdia canadensis* (L.) Nutt. and *Sh. argentea* (Pusch.) Nutt. samples (260 bp and 215 bp, respectively). The PCR products were cloned and sequenced. An analysis of the sequences revealed that intraspecific levels of NTS identity are high (approximately 95–96%) and similar in the *Elaeagnus* L. species. In *Sh. argentea*, this level was slightly lower due to the differences in the poly-T region. Moreover, the intergeneric and intervarietal NTS identity levels were studied and compared. Significant differences between species (except *E. multiflora* Thunb. and *E. umbellata* Thunb.) and genera were found. Herein, a range of the NTS features is discussed. This study is another step in the investigation of the molecular evolution of Elaeagnaceae and may be useful for the development of species-specific DNA markers in this family.

## 1. Introduction

It is well known that the ribosomes of cells in living organisms consist of two subunits, which are named large and small. Ribonucleic acid (RNA) molecules are the important component of both subunits. In cases with all Bacteria, Archaea, and Eucariota, the key role in large subunit formation is played by 5S ribosomal RNA (with the exception of animal and some fungi mitochondria) [1,2]. 5S rRNA is highly conservative, encoded by the 120 bp gene that also has a highly conservative sequence. In the vast majority of seed plants, the 5S rRNA genes are organized as tandem DNA repeats that consist of monomers. Each monomer is composed of the coding 120 bp part and non-transcribed spacer (NTS) [3,4]. In contrast to the coding part, the NTSs may be very different, even among closely related species. Indeed, there are reports about applications of the NTS polymorphism in species-specific DNA marker development [5,6,7].

The *Elaeagnaceae Adans*. family includes three genera: *Elaeagnus*, *Hippophae* L., and *Shepherdia* Nutt. [8]. Plants of these genera grow in temperate zones, mainly in Western Europe, Minor, Central, and Southeast Asia, the Far East, and North America, and less often in the subtropics and tropics. Some species are found in eastern Australia and on the islands of the Sunda Archipelago [9,10,11]. These plants are trees and bushes with shoots and leaves that are often covered with scales or hairs so that they have a silvery green color. Elaeagnaceae plants are classified as phanerophytes, because their buds and shoot tips are high above the soil surface and survive unfavorable times without much protection [12,13]. Moreover, they can grow in regions with poor soil due to the existence of symbiosis with some nitrogen-fixing bacteria in their root nodules [14,15,16]. The *Elaeagnus* genus comprises nearly 100 species of trees and bushes with bisexual flowers. Their shoots are often supplemented with rather long thorns. The most economically valuable species of this genus are *E. angustifolia* L., *E. commutata* Bernh., *E. pungens* Thunb., *E. multiflora*, and *E. umbellata*. These species are grown as ornamental or medicinal plants, and the fruit of *E. angustifolia*, *E. multiflora*, and *E. umbellata* are widely used for food [17]. In this vein, new *Elaeagnus* varieties are bred in many countries. The *Hippophae* and *Shepherdia* species are grown for the same purposes [18,19,20,21]. Unlike the *Elaeagnus* species, they are dioecious plants with male and female flowers in different samples. These species are used for the investigation of plant sex evolution [22,23,24].

The 5S rDNA NTSs of *H. rhamnoides* have previously been sequenced and studied [25]. It was shown that the length of sea buckthorn NTSs is 807 bp. Further analysis has revealed a high level of identity between the rather extended regions in studied NTSs and the sequenced microsatellite loci of *H. rhamnoides* [26], *E. angustifolia* [27], and *Calligonum mongolicum* Turcz. (Polygonaceae) [28]. All of these sequences have a tandem (GA)_10_ motif. To date, this is the only report about NTSs in Elaeagnaceae plants. The NTSs of the *Elaeagnus* and *Shepherdia* species remain unexplored.

In this article, the amplification of the *E. angustifolia*, *E. commutata*, *E. pungens*, *E. multiflora*, *E. umellata*, *Sh. argentea*, and *Sh. canadensis*. 5S rDNA NTSs was carried out. Their sequences were analyzed, compared, and used to find species-specifics polymorphisms.

## 2. Results

### 2.1. Polymerase Chain Reaction (PCR) Amplification of Elaeagnus and Shepherdia 5S rDNA Non-Transcribed Spacers (NTSs)

**Figure 1:**
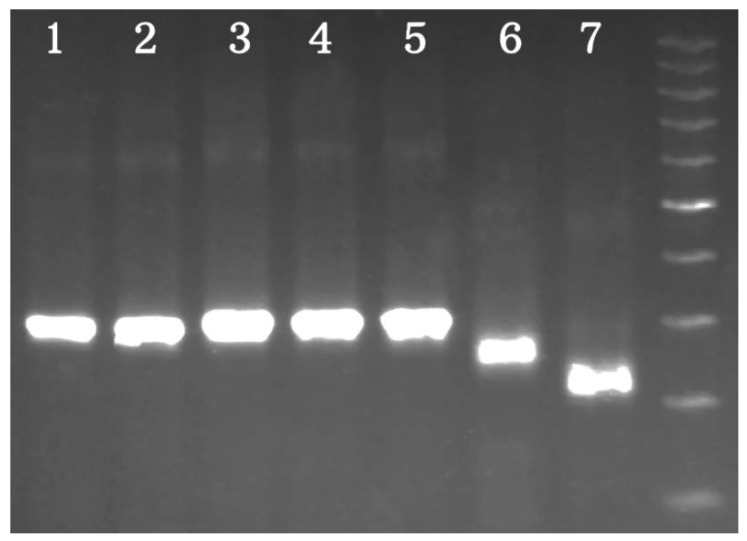
The results of the polymerase chain reaction (PCR) experiments with 5S1/5S2 in the *Elaeagnus* and *Shepherdia* species. The lines correspond to the samples in the follow order: (1) *E. angustifolia*, (2) *E. commutata*, (3) *E. multiflora*, (4) *E. pungens*, (5) *E. umbellata*, (6) *Sh. canadensis*, and (7) *Sh. argentea*. The molecular weight marker is 100 bp.

The amplification of the *E. angustifolia*, *E. commutata*, *E. pungens*, *E. multiflora*, *E. umellata*, *Sh. argentea,* and *Sh. canadensis* 5S rDNA NTSs was carried out with the help of 5S1/5S2 primers, which were designed on the basis of the 5S rRNA encoded *Vitis vinifera* L. conservative sequence [29]. Single PCR fragments were obtained in all samples. All PCR products of the five *Elaeagnus* species had similar lengths near 310 bp. The amplified fragments of *Sh. argentea* and *Sh. canadensis* differed significantly from those of *Elaeagnus* and among themselves in length (they were near 260 bp and 220 bp, respectively). These results are presented in Figure 1.

### 2.2. Sequencing of the PCR Products and Analysis of the Intraspecifc NTS Alignments

**Figure 2:**
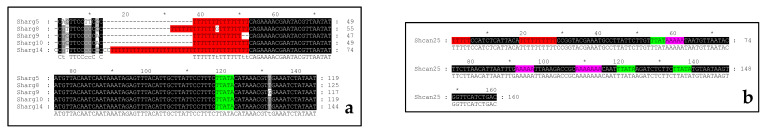
The alignment of *Shepherdia argentea* (**a**) and *Sh. canadensis* (**b**) non-transcribed spacers (NTSs). The poly-T regions are indicated by red. The poly-A region is indicated by pink. The TATA-like motifs are indicated by green.

The 5S1/5S2 PCR products of all of the studied species were cloned into AT-vector and sequenced. Thus, 54 sequences with NTSs were obtained in total: Six from *E. angustifolia*, five from *E. commutata*; five from *E. multiflora*, three from *E. pungens*, 29 from all of the studied varieties of *E. umellata*, five from *Sh. argentea*, and one from *Sh. canadensis* (Table S1). The NTSs from these sequences were compared within each species (or variety for *E. umbellata*) as well as among different species and genera. In the case of all *Elaeagnus* species, the intraspecific level of NTS identity was high and ranged from 95.00 ± 0.71% in *E. pungens* to 96.30 ± 0.59% in *E. commutata* (Table S2). The differences between these values were not statistically significant according to the results of the *t*-tests. However, *Sh. argentea* had some lower values (88.50 ± 2.28%) due to the substantial differences between NTSs in poly-T region (Figure 2a). These values were statistically different from all of the *Elaeagnus* species, except for *E. pungens*.

*Sh. argentea* NTSs consisted of three parts that differ in the level of polymorphism. The first part was a region from the 1st to the 12–15th nucleotides, which mainly included the C and T nucleotides (except the A nucleotides occupying the 2nd position in Sharg5 and the 11th position in Sharg9 and Sharg10) and had the (C)CWYTTCCYYCMC(CC) consensus sequence. The second part was a poly-T region, which was very polymorphic. Sharg5 and Sharg10 had the same formula with 15 T only. In Sharg9 and Sharg14, (T)_13_ and (T)_37_ were observed, respectively. An interesting variant was found in Sharg8, which had the (T)_12_G(T)_8_ formula. The third part (from the 53rd to the 144th position of the alignment) is a highly conservative region, in which only single polymorphic position was observed (132 G/T).

**Figure 3:**
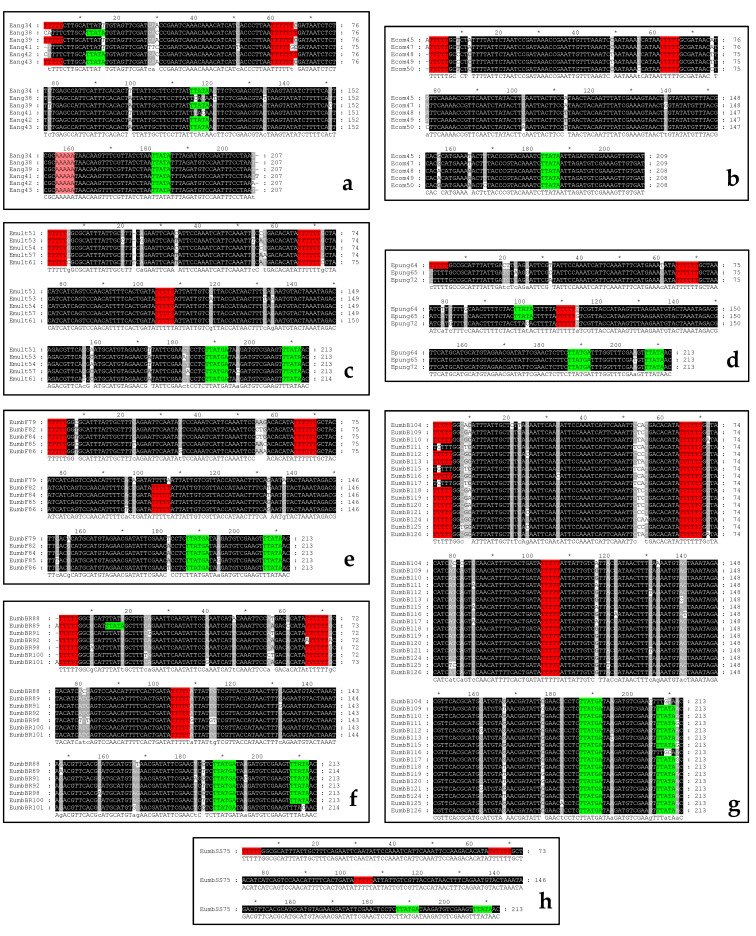
The alignment of the *Elaeagnus angustifolia* (**a**), *E. commutata* (**b**), *E. multiflora* (**c**), *E. pungens* (**d**), *E. umbellata* var. ‘‘Fortunella’’ (**e**), *E. umbellata* var. ‘‘Brilliant Rose’’ (**f**), *E. umbellata* without variety (**g**), and *E. umbellata* var. ‘‘Sweet’n’Sour’’ (**h**) NTSs. The poly-T regions are indicated by red. The poly-A region is indicated by pink. The TATA-like motifs are indicated by green.

The alignment of the *E. angustifolia* NTSs is shown in Figure 3a. Large differences in the NTSs’ length (as in the second part of the *Sh. argentea* NTSs) were not found. All *E. angustifolia* NTSs were 207 bp in length. In the alignment, 18 single nucleotide mutations were identified. Ten C/T transitions and seven transversions were observed (five A/T, one A/C, and one T/G). Thus, more transitions than transversions were revealed in the *E. angustifolia* NTSs. One single nucleotide insertion (T) was found at the 208th position in Eang41. The transcription stop-signal poly-T region without mutations was located from the 1st to the 5th nucleotides in Eang34, Eang39, and Eang43. Another (T)_5_ region was found at the 60th–65th position. Moreover, there was a poly-A region at the 156th–160th position. The TTATA region (181st–185th position or “–27” to “–23” upstream of the coding part) was identified as a TATA-like regulatory element. There were two additional TATA-like motifs at the 7th–11th (in Eang38, Eang42, and Eang43) and the 115th–119th (in Eang34, Eang39, Eang42, and Eang43) positions.

The *E. commutata* NTSs were similar in length (208–209 bp). In the alignment (Figure 3b), 13 single nucleotide substitutions were identified. Ecom45 and Ecom47 had an insertion (A) at the 1st position, and these NTSs were 209 bp in length. Transitions of two types were found (four C/T and one G/A). Transversions were as follows: three A/C, two A/T, and one T/G. Moreover, one multipolymorphic site (A/T/C) was revealed. On the whole, more transversions than transitions were identified in the *E. commutata* NTSs. That is, the picture turned out to be the opposite of that of the *E. angustifolia* NTSs. The poly-T region (a stop-signal for transcription) without mutations was located from the 1st to the 5th nucleotides in Ecom48, Ecom49, and Ecom50. In Ecom45 and Ecom47, it was located at the 2nd–6th position. The next (T)_5_ region was observed at the 62nd–66th position. The TATA-like box with TTATAA (183rd–187th position or “–27” to “–23” upstream of the coding part) was found.

The *E. multiflora* NTSs were slightly longer than the NTSs of *E. angustifolia* or *E. commutata* (Figure 3c). Their lengths were 213–214 bp. The length of Emult61 was 214 bp due to an insertion (T) at the 23rd position. In addition to this mutation, 17 single nucleotide substitutions were seen: nine transitions (two C/G, four A/C, and three A/T); eight transitions (five C/T, and three A/G). The transcription stop-signal poly-T region without mutations locates in the start of all *E. multiflora* NTSs from the 1st to 5th nucleotides. In addition, these NTSs had two other (T)_5_ regions (the 66th–71st and 104th–108th positions, respectively). The TATA-like box was also identified at the 188th–192nd position (“−27”–“−22” upstream to the encoded part), but it had TTATGA formula with G-substitution. Another TATA-like sequence with TTATAA formula was detected at the “−7”–“−2” upstream to the coding part.

For *E. pungens*, three NTSs were sequenced and aligned (Figure 3d). All NTSs were 213 bp in length without indels. The distribution of single nucleotide mutations was the following. There were five C/T transitions and two A/G transitions. The number of and five transversions: one A/C, two A/T and two C/G. The transcription stop-signal (T)_5_ sequence without mutations in the start of NTS was detected in Epung64 only (at the 1st position in other *E. pungens* NTSs the C/T substitution was observed). Two additional (T)_5_ regions were found at the 65th–70th and the 109th–113th positions. The picture of the TATA-like sequences in the *E. pungens* NTSs was the same as in the *E. multiflora* NTSs, but there were found an additional TTATA motif at the 98th–102th position in Epung64 and Epung65.

The NTS sequences of each *E. umbellata* variety were also compared (except “Sweet’n’Sour”, in which one sequence was obtained only). The results are presented in Figure 3e–h and in Table S3. In these NTSs, transversions were slightly more common than transitions. The pictures of the (T)_5_ and TATA-like motif distribution were similar to those in *E. multiflora*, but some *E. umbellata* NTSs had some features. Thus, the second additional (T)_5_ motif in EumbF79 had an A/T substitution at the end, and EumbBR92 had an ATTTT variant of this motif. The transcription stop-signal poly-T in EumbB111, EumbB115, and EumbB117 had a C/T substitution at the 2nd nucleotide position. Moreover, numerous mutations of the “–7” to “–2” TATA-like sequence were detected in EumbB104, EumbB111, EumbB115, EumbB116, and EumbB117. In EumbBR89, there was an additional TTATA motif at the 24th–28th position.

In summary, 137 mutations were detected in all *Elaeagnus* NTS alignments (Table S4): four indels and 133 substitutions. Four substitutions were identified as multipolymorphic. Finally, 129 substitutions were transitions or transversions, transversions were slightly more common. The most frequent mutation was the C/T transition, and the rarest were the T/G and C/G transversions.

An intraspecies nucleotide polymorphism in the *Sh. canadensis* NTS cannot be analyzed, because data for only a single variety were available (Shcan25). However, some features were found in this sequence (Figure 2b). Firstly, it had the transcription stop-signal (T)_5_ at the start, as in most *Elaeagnus* NTSs. This fact distinguished the *Sh. canadensis* NTS from the *Sh. argentea* NTSs. Secondly, Shcan25 had the (T)_10_ region at the same position as the second part of the *Sh. argentea* NTSs. Thirdly, other poly-T sequences were not revealed in Shcan25 as in the *Sh. argentea* NTSs. Lastly, it did not have the second TATA-like box at the “–7” to “–2” position. Thus, Shcan25 combined the features of *Sh. argentea* and *Elaeagnus* NTSs. Moreover, it had its own peculiarities. The most important feature was the presence of the classical TATA-box with the TATAAA formula at the 55th–60th position. Additionally, there was one additional TATA-like motif at the 119th–125th position (TTATAAG). Finally, Shcan25 had three poly-A regions at the 58th–62nd, 92nd–96th, and 108th–114th positions.

### 2.3. Interspecific, Intervarietal, and Intrageneric Level of NTS Identity in the Studied Elaeagnaceae Plants

In the next step of the investigation, all NTS sequences were aligned and identity values were calculated (Table S5). The intervarietal and interspecific identity values were computed for further analysis by *t*-tests. Thereby, collation of the intraspecific (intravarietal) and interspecific levels of identity was carried out (Table S6). The lowest level of interspecific identity was observed in the *Sh. argenteae*/*Sh. canadensis* pair (45.80 ± 1.75%). In *Elaeagnus* spp., the lowest level of interspecific identity was observed in the *E. angustifolia*/*E. umbellata* var. “Sweet’n’Sour” pair (55.17 ± 0.18%). The highest value of this indicator (97.20 ± 0.55%) was in the *E. multiflora*/*E. umbellata* var. “Sweet’n’Sour” pair. In general, *E. multiflora* showed high values in the pairs with all varieties of *E. umbellata*. These values did not have any statistically valuable differences compared to the *E. multiflora* intraspecific level of identity. *E. pungens* also showed high values in the pairs with *E. multiflora* and *E. umbellata* (84–86%), but these values were reliably lower than their intraspecific values. Other pairs demonstrated a near 60% level of interspecific identity, which reliably differed from the corresponding intraspecific values according to the *t*-tests. The levels of intervarietal identity were high (from 95.31 ± 0.12% to 97.43 ± 0.32%) and some values were higher than the corresponding intravarietal identity values. However, some differences were not statistically confirmed, since the empirical values of the *t*-tests fell into an uncertainty zone.

Similar comparative analyses were carried out in the two investigations of the intergeneric level of identity (*Sh. argentea*/*Elaeagnus* spp. and *Sh. canadensis*/*Elaeagnus* spp.). The results of these investigations are presented in Tables S7 and S8. In each line of the matrices obtained, the comparisons of the intergeneric and interspecific (from Table S5) values were conducted by *t*-tests. All differences were identified as statistically significant. The intergeneric values with *Sh. argentea* were low (from 30.60 ± 0.35% to 33.76 ± 0.28%). In the case of *Sh. canadensis*, the intergeneric values were higher, but they could not be assessed as high (from 41.43 ± 0.32% to 45.67 ± 0.41%).

## 3. Discussion

In this article, 5S rDNA NTS sequences were presented and analyzed for the first time in five *Elaeagnus* species and two *Shepherdia* species from the Elaeagnaceae family. The 5S rDNA in these species is obviously organized according to the classical model, with alternations of the conservative coding and variable non-coding parts [30]. This conclusion was indirectly confirmed by the successful PCR in all of the studied samples using universal primers designed for the conserved coding region [29]. The electrophoresis detection showed that the patterns of all of the PCR products consisted of single fragments. Such an electrophoretic pattern was previously observed in other experiments with the same (or similar) universal primers. For example, this picture has been revealed in *H. rhamnoides*, *Petrosimonia oppositifolia* (Palas) Litvinov, *P. glaucescens* (Bunge) Iljin, *Populus suaveolens* Fisch., *Populus koreana* Rehder., and other species [25,31,32,33]. A radically different pattern was observed in species of a hybrid or polyploid origin. In such cases, the electrophoresis discovers two or more fragments. These results have been described in *Petrosimonia litwinowii* Korsh., *Populus deltoides* Bartr. ex Marshall, *Populus alba* L., *Populus ×rasumowskyana* (R.I. Schrod. ex Regel) C.K. Schneid., and *Atropa belladonna* L. Poaceae Barnhart. spp. [31,34,35,36,37,38]. Thus, it can be assumed that the *Elaeagnus* and *Shepherdia* species are most likely not of a hybrid or polyploid origin.

As a potentially useful result, differences in the length of the PCR products between *Sh. argentea*, *Sh. canadensis,* and *Elaeagnus* spp. were detected. The corresponding single fragments of 220, 260, and 310 bp in length were clearly distinguishable in the electrophoresis picture. In addition, they can be even more distinguishable with the ~900 bp PCR product included the *H. rhamnoides* NTSs [25]. Thus, these differences can be used at the first stage of Elaeagnaceae plant identification, when species-specific morphological characters do not allow exact identification (for example, in the leafless period or in homogenized samples). However, the *Elaeagnus* species cannot be identified in this way due to a high similarity in the length of their PCR products. In this case, PCR products require sequencing and comparison with the sequences presented in this article. Similarity in length is not rare among NTSs of closely related species. Therefore, the previously sequenced NTSs of *P. suaveolens* and *P. koreana* were shown to have similar lengths and polymorphic regions [32,33].

In this work, 54 sequences of the Elaeagnaceae NTSs were obtained. The intraspecific alignments required finding some interesting features in these NTSs. For example, one of such features was poly-T regions in different places of the NTSs. All *Elaeagnus* and *Sh. canadensis* species had the (T)_5_ region at the start of their NTSs (but different single nucleotide mutations were found in some NTSs). This feature was also detected in the previously described NTSs of *Vitis vinifera*, *A. belladonna*, *Phytolacca Americana* L., *Thinopyrum intermedium* (Host) Barkworth and D.R. Dewey, *Lens culinaris* Medic., and others [29,37,39,40,41]. However, *Sh. argentea* had a C/T-rich part at the start of its NTSs instead of the (T)_5_ region and the poly-T stretch after the 12th–15th nucleotides only. Similar variants at the start of the NTSs have also found in other plants, such as *Populus euphratica* Olivier [36]. In the *Elaeagnus* NTSs, two additional poly-T motifs were revealed. Such motifs are far from uncommon among NTSs. In a range of balsamic poplar species, there are many poly-T or poly-A regions, and their NTSs are often AT-rich [36].

The next important feature of the NTSs was the presence of TATA-like boxes in the upstream region relative to the coding part of the 5S rDNA. Sometimes, even several TATA-like sequences are interspersed into one NTS. Such incidents have been described in different organisms: *Engistomops* Jiménez De La Espada spp., *Donax* L. spp., *Erythroculter ilishaeformis* Bleeker, *Populus fremontii* S. Watson, etc. [36,42,43,44]. In the studied Elaeagnaceae species, there was one TATA-like motif in the NTSs of *Sh. argentea*, two in *Elaeagnus* spp., and three in the *Sh. canadensis*. The Shcan25 TATA-box from the 55th–60th position had the classical TATAAA formula, and the others were slightly modified. On the whole, the modified variants of the TATA-box come across more often. For example, TTATAAG (119th–125th position of the *Sh. canadensis* NTS) has also been found in a wide range of poplars (*Populus ciliata* Wall. ex Royle, *P. trichocarpa* (Torr. and A. Gray ex Hook.) Brayshaw, *P. lasiocarpa* Oliv., *P. simonii* Carriére, *P. deltoides*, *P. yunnanensis* Dode, etc.) [34,36]. TATATA, TGATATA, and TATTTA occur in the NTSs of different *Donax* species, TTATAT in *Engistomops* spp., TTATGTA in *H. rhamnoides* and most of the other variants [25,42,43]. Assessing the functionality of these modified TATA-boxes as regulatory elements (TATA-box is a signal sequence for RNA polymerase II/III [45]) is difficult, and a lot of in vitro translation experiments are required.

In all *Elaeagnus* intraspecific NTS alignments, different mutations were counted. The observed mutations were single nucleotide substitutions or indels without regularity of their locations. Numerous reports have provided information regarding a similar picture of NTS mutations in other organisms [29,37,42]. Authors’ attention is often drawn to the ratio of transitions to transversions. Thus, there is an opinion that transitions are usually more common than transversions in the coding regions of the genome [46,47]. However, NTSs are not coding regions and this rule may not work. In this article, it was found that the ratio of transitions to transversions was nearly 1:1 (the prevalence of transversions was small). Studying the substitutions in the long variants of *A. belladonna* NTSs, Volkov et al. observed the same ratio. However, the intermediate and short variants had more transitions [37]. Moreover, Maughan et al. reported different ratios of transitions to transversions in two NTS classes in *Chenopodium quinoa* C.L. Willdenow (Willd.) (transitions predominated in class I, and transversions were more common in class II) [41]. In sum, there are different cases that provide different ratios of transitions to transversions among different NTSs.

The investigations of the intraspecific, interspecific, intervarietal, and intergeneric identity in the studied Elaeagnaceae plants found that the intraspecific level of NTS identity was high (from 95.00 ± 0.71% to 96.30 ± 0.59%) in *Elaeagnus* spp. In the case with the *A. belladonna* long variant of NTSs, the sequence identity was also high (from 96.40% to 99.60%) [37]. In addition, three species of the *Rosa* L. genus had the following values of this indicator: In *R. wichurana* Crép., from 91.70% to 95.40%; in *R. rugosa* Thunb., 99.10%; in *R. nitida* Willd., from 97.90% to 99.50% [48]. The deviation from 100% in all cases was due to a small number of single nucleotide changes. The sequences of the *Sh. argentea* NTS had more serious differences in the poly-T part and the intraspecific level of their identity was significantly lower (88.50 ± 2.28%). The differences observed in the poly-T regions could be for different reasons. It is possible that some of them are associated with errors in the Taq-polymerase work during PCR because poly-A/T stretches are a difficult template [49,50]. However, it is equally possible that this part of the *Sh. argentea* NTSs behaves like a microsatellite, and that the differences in the length of the poly-T motifs were caused by errors in replication. In general, microsatellites were previously found in the NTSs of *P. deltoides*, *H. rhamnoides*, *L. culinaris*, some species of fish, etc. [25,34,41]. It is interesting to note that the (GA)_9_ microsatellite of the *H. rhamnoides* NTSs was absent in the *Elaeagnus* and *Shepherdia* NTSs studied.

Some interspecific and all intervarietal values of the NTS identity in the *Elaeagnus* genus were similar to the values of the intraspecific identity. These results were obtained in the comparison between the *E. multiflora* and *E. umbellata* NTSs. Thus, these results provide a reason to consider these species names synonymous. Such a concept has been expressed previously, i.e., by Handell-Mazzetti [51]. The intervarietal values of identity in several *E. umbellata* varieties were high (from 95.31 ± 0.12% to 97.43 ± 0.32%). Therefore, NTSs are not suitable for creating variety-specific molecular markers, at least not in this case. The other interspecific values of NTS identity among *Elaeagnus* spp. ranged from 55.17 ± 0.18% to 86%. The values of this indicator in such plants as *Nicotiana* L. spp. (nearly 80%), *Astrgalus* L. spp. (nearly 70%), and *Rosa* spp. (from 52.8% to 57.6%) are also within this range [48,52,53]; however, the value of the *Sh. argenteae*/*Sh. canadensis* pair (45.80 ± 1.75%) was not. The presence of large polymorphisms in these NTSs could be useful, for example, in species-specific marker development (as there are difficulties in the differentiation of these *Shepherdia* species based on their morphological characteristics, especially during the juvenile and leafless periods). Finally, the intergeneric values of the NTS identity between two *Shepherdia* and five *Elaeagnus* species were low and ranged from 30.60 ± 0.35% to 45.67 ± 0.41%. Thus, three ranges of NTS identity can be indicated in the case with the studied Elaeagnaceae spp.: 30–45% for the intergeneric, 45–86% for the interspecific, and 95–97% for the intraspecific level.

The results obtained may be interesting for the wide range of specialists that study the organization of 5S rDNA. Furthermore, this study is another step in the investigation of the molecular evolution of Elaeagnaceae and may be useful for the development of species-specific DNA markers in this family.

## 4. Materials and Methods

### 4.1. Plant Material and DNA Isolation

In this work, a collection of *Elaeagnus* spp. and *Shepherdia* spp. samples were created (Table S9). The *E. angustifolia*, *E. commutata*, *E. multiflora,* and *E. umbellata* var. “Sweet’n’Sour” trees were grown in open ground in KIZ “Allea”, Kievsky village, Moscow, Russia. The *E. umbellata* var. “Pointilla Fortunella”, “Brilliant Rose” bushes, *E. umbellata* sample without the variety from the “Sad pochtoj” nursery (cloned from non-identified cuttings, Bulgaria), and *E. pungens* var. “Maculata” were grown in a greenhouse in the All-Russia Research Institute of Agricultural Biotechnology, Timiryazevskaya 42, Moscow, Russia. The leaf tissue samples of *Sh. argentea* and *Sh. canadensis* were received as dried material from the Arnold Arboretum of Harvard University (Boston, MA, USA) and Botanic Garden Meise (Meise, Belgium), respectively. DNA from young, fresh, and dried leaves was isolated according to the Doyle and Doyle CTAB protocol with some modifications [54,55]. DNA samples were equalized in concentration, aliquoted, and stored at –20 °C.

### 4.2. The PCR Experiments, Electrophoresis and Sequencing

Amplification of the 5S rDNA fragments was carried out with the 5S1/5S2 primers designed by Falistocco et al. [29]. The primers were synthesized by ZAO “Synthol” (Moscow, Russia). PCR was performed under the following conditions: 94 °C for 3 min; 35 cycles of 94 °C for 20 s, 60 °C for 20 s, 72 °C for 20 s; 72 °C for 10 min. The PCR mix consisted of approximately 10 ng of genomic DNA, 2.5 U of Taq-polymerase (ZAO “Sibenzyme”, Novosibirsk, Russia), 1× SE-buffer, 2.5 mM of MgCl_2_, 100 µM of each dNTP, and 0.25 µM of the forward and reverse primers and ddH2O. The PCR products were detected by electrophoresis on 2.5% agarose gel at 10 V/cm in 0.5 M of TBE buffer using a Sub-Cell Model 192 camera (Bio-Rad, Hercules, CA, USA) and photographed using the gel documentation system GelDoc XR Plus (Bio-Rad, Hercules, CA, USA). Purification of the PCR products was carried out with the “CleanMag” Kit ZAO “Evrogen” (Moscow, Russia). The purified amplicons were cloned to the AT-vector (pAL2-T Kan, ZAO “Evrogen”, Moscow, Russia). Clones with the target insert were found by white-blue selection and PCR testing with the M13 standard primers. Sequencing of M13 PCR products was performed by ZAO “Evrogen” (Moscow, Russia).

### 4.3. Analysis of the Sequences and Statistical Tests

The sequences were processed in the GenDoc program (coding parts were cut) [56], and the NTSs were collected and submitted to GenBank (Table S1). The alignments and calculations of the NTS identity values were also carried out in GenDoc. The calculation of the average NTS identity values (of the intraspecific, interspecific, and intergeneric levels) and the standard errors was carried out using an online calculator (https://medstatistic.ru/calculators/calcvaries.html). Comparison of the average values was performed using Student *t*-tests with an online calculator (https://www.psychol-ok.ru/statistics/student/) for unrelated samples.

## Data Availability

*Shepherdia* spp. samples were used in this study. The *Sh. argentea* dried leaves were kindly provided by Kathryn Richardson (AA#102-77*A sample of Arnold Arboretum, The Harvard University, USA). The *Sh. canadensis* dried leaves were kindly provided by Kenneth Bauters (*19801643-I34ZZ sample of Botanic Garden Meise, Belgium).

## Supplementary Materials

The following are available online at https://www.mdpi.com/2223-7747/10/1/4/s1: Table S1. The sequences of Elaeagnaceae spp. non-transcribed spacers (NTSs) and their characteristics; Table S2. Comparisons of the average values of intraspecific NTS identity by *t*-tests; Table S3. Mutations in the *Elaeagnus umbellata* NTSs; Table S4. Mutations in the *Elaeagnus* spp. NTSs; Table S5. The values of NTS identity at the intraspecific (or intravarietal), interspecific, intervarietal and intergeneric levels; Table S6. A comparative analysis of the average levels of interspecies (intervariety for *Elaeagnus umbellata*) identity by *t*-tests; Table S7. A comparative analysis of the average levels of interspecies (intervariety for *Elaeagnus umbellata*) and intergeneric (between *Shepherdia argentea* and *Elaeagnus* spp.) identity by *t*-tests; Table S8. A comparative analysis of the average levels of interspecies (intervariety for *Elaeagnus umbellata*) and intergeneric (between *Shepherdia canadensis* and *Elaeagnus* spp.) identity by *t*-tests; Table S9. The studied plants of the *Elaeagnus* and *Shepherdia* species.
