# Supplementary material for: A Comparative Study of 5S rDNA Non-Transcribed Spacers in Elaeagnaceae Species
Source: Plants (Basel). 2020 Dec 23;10(1):4. doi: 10.3390/plants10010004 (PMC7822202; doi:10.3390/plants10010004)
Supplement: Supplementary file 1 [file plants-10-00004-s001.zip › Table S7.pdf]

Table S7. A comparative analysis of the average levels of interspecies (intervariety for *Elaeagnus umbellata*) and intergeneric (between *Shepherdia argentea* and *Elaeagnus* spp.) identity by *t*-tests.

	Shepherdia argentea	*Elaeagnus angustifolia*	*Elaeagnus commutata*	*Elaeagnus multiflora*	*Elaeagnus pungens*	*Elaeagnus umbellata* Fortunella	*Elaeagnus umbellata* Brilliant Rose	*Elaeagnus umbellata* B	*Elaeagnus umbellata* Sweet'n'Sour
Elaeagnus angustifolia	30,60±0,47	96,07±0,47	60,83±0,25	57,03±0,26	58,67±0,20	56,87±0,18	56,81±0,19	55,57±0,11	55,17±0,18
Elaeagnus commutata	31,32±0,49	60,83±0,25	96,30±0,59	60,56±0,15	60,93±0,19	62,12±0,18	61,29±0,10	61,13±0,07	61,60±0,27
Elaeagnus multiflora	31,12±0,25	57,03±0,26	60,56±0,15	95,70±0,59	84,50±0,18	95,56±0,26	95,54±0,20	95,53±0,12	97,20±055
Elaeagnus pungens	33,67±0,35	58,67±0,20	60,93±0,19	84,50±0,18	95,00±0,71	84,00±0,00	84,95±0,18	84,58±0,11	86,00±0,00
Elaeagnus umbellata Fortunella	33,76±0,28	56,87±0,18	62,12±0,18	95,56±0,26	84,00±0,00	96,10±0,48	95,51±0,15	95,31±0,12	97,40±0,27
Elaeagnus umbellata Brilliant Rose	32,46±0,35	56,81±0,19	61,29±0,10	95,54±0,20	84,95±0,18	95,51±0,15	95,48±0,23	95,23±0,13	97,43±0,32
Elaeagnus umbellata B	30,94±0,19	55,57±0,11	61,13±0,07	95,53±0,12	84,58±0,11	95,31±0,12	95,23±0,13	95,78±0,17	96,94±0,96
Elaeagnus umbellata Sweet'n'Sour	33,40±0,57	55,17±0,18	61,60±0,27	97,20±055	86,00±0,00	97,40±0,27	97,43±0,32	96,94±0,96	

[red] - average value of intergeneric identity, which is compared with correspond intraspecies values in line

[green] - differences are statistically significant

The empirical values of t-criteria									
	Shepherdia argentea	*Elaeagnus angustifolia*	*Elaeagnus commutata*	*Elaeagnus multiflora*	*Elaeagnus pungens*	*Elaeagnus umbellata* Fortunella	*Elaeagnus umbellata* Brilliant Rose	*Elaeagnus umbellata* B	*Elaeagnus umbellata* Sweet'n'Sour
Elaeagnus angustifolia		109,4	70,3	60,1	57,5	66,4	70	88,3	30,3
Elaeagnus commutata		57,3	77,5	59,7	46,5	60,4	71	103	27,8
Elaeagnus multiflora		69,8	105,1	123,2	154,4	184,1	200,5	245,9	110,9
Elaeagnus pungens		67	73,7	138,2	77,1	152,5	146,8	188,2	68,5
Elaeagnus umbellata Fortunella		72,4	88,6	167	139	118,3	209,8	225,8	99,5
Elaeagnus umbellata Brilliant Rose		63,4	77,9	153,9	109,2	165,9	128	203,8	79,9
Elaeagnus umbellata B		115,3	149,9	293,6	205,2	292,6	286,4	248,1	149,1
Elaeagnus umbellata Sweet'n'Sour		43,9	50,4	91,1	77,4	114,3	115,8	120,5	

The critical values of t-criteria									
	Shepherdia argentea	*Elaeagnus angustifolia*	*Elaeagnus commutata*	*Elaeagnus multiflora*	*Elaeagnus pungens*	*Elaeagnus umbellata* Fortunella	*Elaeagnus umbellata* Brilliant Rose	*Elaeagnus umbellata* B	*Elaeagnus umbellata* Sweet'n'Sour
Elaeagnus angustifolia		2,02	2	2	2,01	2	1,99	1,97	2,03
Elaeagnus commutata		2	2,04	2,01	2,02	2,01	2	2,61	2,05
Elaeagnus multiflora		2	2,01	2,04	2,02	2,01	2,66	1,97	2,05
Elaeagnus pungens		2,04	2,05	2,05	2,12	2,05	2,03	1,99	2,15
Elaeagnus umbellata Fortunella		2	2,01	2,01	2,02	2,04	2	1,97	2,05
Elaeagnus umbellata Brilliant Rose		1,99	1,99	1,99	2	1,99	2	1,97	2,02
Elaeagnus umbellata B		1,97	1,97	1,97	1,97	1,97	1,97	1,97	1,98
Elaeagnus umbellata Sweet'n'Sour		2,26	2,31	2,31	2,45	2,31	2,33	2,09	
