# Supplementary material for: A Comparative Study of 5S rDNA Non-Transcribed Spacers in Elaeagnaceae Species
Source: Plants (Basel). 2020 Dec 23;10(1):4. doi: 10.3390/plants10010004 (PMC7822202; doi:10.3390/plants10010004)
Supplement: Supplementary file 1 [file plants-10-00004-s001.zip › Table S8.pdf]

Table S8. A comparative analysis of the average levels of interspecies (intervariety for *Elaeagnus umbellata*) and intergeneric (between *Shepherdia canadensis* and *Elaeagnus* spp.) identity by *t*-tests.

	Shepherdia canadensis	*Elaeagnus angustifolia*	*Elaeagnus commutata*	*Elaeagnus multiflora*	*Elaeagnus pungens*	*Elaeagnus umbellata* Fortunella	*Elaeagnus umbellata* Brilliant Rose	*Elaeagnus umbellata* B	*Elaeagnus umbellata* Sweet'n'Sour
Elaeagnus angustifolia	41,67±0,23	96,07±0,47	60,83±0,25	57,03±0,26	58,67±0,20	56,87±0,18	56,81±0,19	55,57±0,11	55,17±0,18
Elaeagnus commutata	41,80±0,22	60,83±0,25	96,30±0,59	60,56±0,15	60,93±0,19	62,12±0,18	61,29±0,10	61,13±0,07	61,60±0,27
Elaeagnus multiflora	41,80±0,22	57,03±0,26	60,56±0,15	95,70±0,59	84,50±0,18	95,56±0,26	95,54±0,20	95,53±0,12	97,20±055
Elaeagnus pungens	45,67±0,41	58,67±0,20	60,93±0,19	84,50±0,18	95,00±0,71	84,00±0,00	84,95±0,18	84,58±0,11	86,00±0,00
Elaeagnus umbellata Fortunella	42,40±0,27	56,87±0,18	62,12±0,18	95,56±0,26	84,00±0,00	96,10±0,48	95,51±0,15	95,31±0,12	97,40±0,27
Elaeagnus umbellata Brilliant Rose	41,43±0,32	56,81±0,19	61,29±0,10	95,54±0,20	84,95±0,18	95,51±0,15	95,48±0,23	95,23±0,13	97,43±0,32
Elaeagnus umbellata B	42,06±0,11	55,57±0,11	61,13±0,07	95,53±0,12	84,58±0,11	95,31±0,12	95,23±0,13	95,78±0,17	96,94±0,96
Elaeagnus umbellata Sweet'n'Sour	42,00±00	55,17±0,18	61,60±0,27	97,20±055	86,00±0,00	97,40±0,27	97,43±0,32	96,94±0,96	

[red box] - average value of intergeneric identity, which is compared with correspond intraspecies values in line

[green box] - differences are statistically significant

The empirical values of t-criteria									
	Shepherdia canadensis	*Elaeagnus angustifolia*	*Elaeagnus commutata*	*Elaeagnus multiflora*	*Elaeagnus pungens*	*Elaeagnus umbellata* Fortunella	*Elaeagnus umbellata* Brilliant Rose	*Elaeagnus umbellata* B	*Elaeagnus umbellata* Sweet'n'Sour
Elaeagnus angustifolia		73,8	33,5	25	46,3	36,3	29,9	30,6	51,9
Elaeagnus commutata		30,5	66,7	56,3	56,5	49,8	67,3	70,7	63,9
Elaeagnus multiflora		22,7	56,3	66	131,7	93	96,6	106,9	106,5
Elaeagnus pungens		25,5	35	93,1	74,7	269,9	80,9	86	122,2
Elaeagnus umbellata Fortunella		31,6	47,8	91,5	312	78,8	129,5	104,4	161,8
Elaeagnus umbellata Brilliant Rose		32,2	73,9	112,1	124,5	149,7	126,4	100,8	133,3
Elaeagnus umbellata B		48,2	120,6	188,5	209,6	186,5	150,9	113,8	203,3
Elaeagnus umbellata Sweet'n'Sour		29,9	32,7	46	54,3	92,3	65,9	53,4	

The critical values of t-criteria									
	Shepherdia canadensis	*Elaeagnus angustifolia*	*Elaeagnus commutata*	*Elaeagnus multiflora*	*Elaeagnus pungens*	*Elaeagnus umbellata* Fortunella	*Elaeagnus umbellata* Brilliant Rose	*Elaeagnus umbellata* B	*Elaeagnus umbellata* Sweet'n'Sour
Elaeagnus angustifolia		2,09	2,03	2,03	2,07	2,03	2,01	1,98	2,23
Elaeagnus commutata		2,04	2,16	2,05	2,1	2,05	2,02	1,99	2,31
Elaeagnus multiflora		2,04	2,05	2,16	2,1	2,05	2,02	1,99	2,31
Elaeagnus pungens		2,09	2,12	2,12	2,78	2,12	2,07	2	2,78
Elaeagnus umbellata Fortunella		2,03	2,05	2,05	2,1	2,16	2,02	1,99	2,31
Elaeagnus umbellata Brilliant Rose		2,01	2,02	2,02	2,06	2,02	2,06	1,97	2,18
Elaeagnus umbellata B		1,97	1,98	1,98	1,99	1,98	1,97	1,97	2,04
Elaeagnus umbellata Sweet'n'Sour		2,57	2,78	2,78	2,78	2,78	2,45	2,13	
