# Supplementary material for: A Comparative Study of 5S rDNA Non-Transcribed Spacers in Elaeagnaceae Species
Source: Plants (Basel). 2020 Dec 23;10(1):4. doi: 10.3390/plants10010004 (PMC7822202; doi:10.3390/plants10010004)
Supplement: Supplementary file 1 [file plants-10-00004-s001.zip › Table S9.pdf]

Table S9. The studied plants of *Elaeagnus* and *Shepherdia* species.

Species	Variety	Code	Origin	Coordinates or greenhouse/dried material
Elaeagnus angustifolia		Eang	‘’Martin Sad’’ nursery, Mytischi, Russia	55°41’49.65’’ 36°84’83.48’’
Elaeagnus commutata		Ecom	Street planting in ‘’KIZ Allea’’, Kievsky village, Moscow, Russia	55°41’69.27’’ 36°84’76.71’’
Elaeagnus multiflora		Emult	‘’Nara-Sad’’ nursery, Naro-Fominsk, Russia	55°41’48.48’’ 36°84’79.92’’
Elaeagnus pungens	‘’Maculata’’	Epung	‘’Opt-khoz’’ nursery, Ozery, Russia	greenhouse
Elaeagnus umbellata	‘’Pointilla Sweet’n’Sour’’	EumbSS	‘’Darvin’’ nursery, Moscow, Russia	55°41’48.64’’ 36°84’85.38’’
	‘’Pointilla Fortunella’’	EumbF	‘’Sad pochtoj’’ nursery, St. Petersburg, Russia	greenhouse
	‘’Brilliant Rose’’	EumbBR	‘’Sad pochtoj’’ nursery, St. Petersburg, Russia	greenhouse
	without variety	EumbB	‘’Sad pochtoj’’ nursey, St. Petersburg, Russia	greenhouse
Shepherdia argentea		Sharg (AA#102-77*A)	Arnold Arboretum, The Harvard University, USA	dried material
Shepherdia canadensis		Shcan (*19801643-I34ZZ)	Botanic Garden Meise, Nieuwelaan, 38, 1860 Meise, Belgium	dried material
