# Supplementary material for: A Comparative Study of 5S rDNA Non-Transcribed Spacers in Elaeagnaceae Species
Source: Plants (Basel). 2020 Dec 23;10(1):4. doi: 10.3390/plants10010004 (PMC7822202; doi:10.3390/plants10010004)
Supplement: Supplementary file 1 [file plants-10-00004-s001.zip › Table S1.pdf]

Table S1. The sequences of NTSs and their characteristics.

Species	Variety	Code	Sequence	Length, bp	GenBank
E. angustifolia		Eang34	TTTTTCTTGCATTATTTGTAGTTCGATCAACCGAATCAAACAAACATCATCACCCTTAAT TTTTTCGATAATCTCTTCTGAGCCATTCATTTCACACTTTTATTGCTTCCTTATTTATAA CTTCTCGAACGTACTAAGTATATCTTTTCATTCGCAAAAATAACAAGTTTCGTTATCTAA TTATATTTAGATGTCCAATTTCCTAAT	207	MW288958
		Eang38	CATTTCTTGCATTATATGTAGTTCGATCACCCGAATCAAACAAACATCATCACCCTTAAT TTTTTTGATAATCTCTTCTGAGCCATTCATTTCACACTATTATTGCTTCCTTATTCATAA TTTCTCGAACGTATTAAGTATATCTTTTCATTCGCAAAAATAACAAGTTTCGTTATCTAA TTATATTTAGATGTCCAATTTCCTAAT	207	MW288959
		Eang39	TTTTTCTTGCATTATTTGTAGTTCGATCAACCGAATCAAACAAACATCATCACCCTTAAT TTTTTCGATAATCTCTTTTGAGCCATTCATTTCACACTATTATTGCTTCCTTATTTATAA CTTCTCGAACGTACTAAGTATATCTTTTCACTCGCAAAAATAACAAGTTTCGTTATCTAA TTATATTTAGATGTCCAATTTCCTAAT	207	MW288960
		Eang41	TTTTCTTGCATTATTTGTAGTTCGATTTCCCGAATCAAACAAACATCATTACCCTTAATT TTTGCGATAATCTCTTCTGAGCCATTCATTTCACACTTTTATTGCTTCCTTATTTACAAC TTCTCGAACGTACTAAGTATATCTTTTCATTCGCAAAAATAACAAGTTTCGTTATCTAAT TATATTTAGATGTCCAATTTCCTAATT	207	MW288961
		Eang42	CTTTTCTTGCATTATATGTAGTTCGATCACCCGAATCAAACAAACATCATCACCCTTAAT TTTTTTGATAATCTCTTCTGAGCCATTCATTTCACACTATTATTGCTTCCTTATTTATAA CTTCTCGAACGTACTAAGTATATCTTTTCATTCGCAAAAATAACAAGTTTCGTTATCTAA TTATATTTAGATGTCCAATTTCCTAAA	207	MW288962
		Eang43	TTTTTCTTGCATTATATGTAGTTCGATCAACCGAATCAAACAAACATCATCACCCTTAAT TTTTTCGATAATCTCTTCTGAGCCATTCATTTCACACTATTATTGCTTCCTTATTTATAA CTTCTCGAACGTACTAAGTATATCTTTTCATTCGCAAAAATAACAAGTTTCGTTATCTAA TTATATTTAGATGTCCAATTTCCTAAT	207	MW288963
E. commutata		Ecom45	ATTTTTGCTCTATTTTATTCTAATCCGATAAACCGAATTGTTTAAATCCAATAAATCATA ATTTTTGCGATAACATATTCAAAACCGTTCAATCTATACTTCAATTACTTCCATAACTAC AATTTATCGAAAGTAACTTGTATATGTTTACGCACGCATGAAATACTTTACCCGTACAAA TCTTATAATTAGATGTCGAAAGTTGTGAT	209	MW288964
		Ecom47	ATTTTTGCTCTATTTTATTCTAATCCGATAAACCGAATTGTTTAAATCCAATAAATCATA ATTTTTGCGATAACATATTCAAAACCGTTCAATCTATACTTCAATTACTTCCATAACTAC	209	MW288965

			AATTTATCGAAAGTAACTGGTATATGTTTACGCACGCATGAAATACTTTACCCGTACAAA TCTTATAATTAGATGTCGAAAGTTGTGAT		
		Ecom48	TTTTTGCCCTCTTTTATTCTAATCCGATAAACCGAATTGTTTAAATCTAATAAATCATAA TTTTTGCGATAACTTATTCAAAACCGTTCAATCTATACTTCAATTACTTCCTTAACTACA ATTTATCGAAAGTAACTTGTATATGTTTACGCACACATGAAAAACTTTACCCGTACAAAT CTTATAATTAGATGTCGAAAGTTGTGAT	208	MW288966
		Ecom49	TTTTTGCCCTCTTTTATTCTAATCCGATAAACCGAATTGTTTAAATCTAATAAATCATAA TTTTTGCGATAACTTATTCAAAACCGTTCAATCTATACTTCAATTACTTCCTTAACTACA ATTTATCGAAAGTAACTTGTATATGTTTACGCACACATGAAAAACTTTACCCGTACAAAT CTTATAATTAGATGTCGAAAGTTGTGAT	208	MW288967
		Ecom50	TTTTTGCTCTCTTTTATTCTAATCCGATAAACCGAATTGTTTAAATCCAATAAACCATAA TTTTTGCGATAACCTCTTCAAAACCGTTCAATCTATACTTAAATTACTTCCTTAACTACA ATTTATCGAAAGTAACTTGTATATGTTTACGCACGCATGAAAAACTCTACCCGTACAAAT CTTATAATTAGATGTCGAAAGTTGTGAT	208	MW288968
E. multiflora		Emult51	TTTTTGGCGCATTTATTGCTTTCAGAATTCAACATTCCAAATCATTCAAATTCCCTGACA CATATTTTTTGCTACATCATCAGTCCAACATTTTCACTGATATTTTTATTATTGTCGTTA CCATAACTTTCAGAATGTACTAAATAGACGTTCACGAATGCATGTAGAACGTTATTCGAA CTCCTCTTATGATAAGATGTCGAAGTTTATAAC	213	MW288969
		Emult53	TTTTTGGCGCATTTATTGCCTTCGGAATTCAATATTCCAAATCATTCAAATTTCATGACA CATATTTTTTGCTACATCATCAGTCCAACATTTTCACTGATATTTTTATTATTGTCATTA CCATAACTTTTAAAATGTACTAAATAGACGTTCACGCATGCATGTAGAACGATATTCGAA ATCCTCTTATGATAAGATGTCGAAGTTTATAAC	213	MW288970
		Emult54	TTTTTGGCGCATTTATTGCTTTCAGAATTCAACATTCCAAATCATTCAAATTCCCTGACA CATATTTTTTGCTACATCATCAGTCCAACATTTTCACTGATATTTTTATTATTGTCGTTA CCATAACTTTCAGAATGTACTAAATAGACGTTCACGAATGCATGTAGAACGTTATTCGAA CTCCTCTTATGATAAGATGTCGAAGTTTATAAC	213	MW288971
		Emult57	TTTTTGGCGCATTTATTGCTTTCAGAATTCAATATTCCAAATCATTCAAATTCCAAGACA CATATTTTTTGCTACATCATCAGTCCAACATTTTCACTGATATTTTTATTATTGTCGTTA CCATAACTTTCAGAATGTACTAAATAGACGTTCACGCATGCATGTAGAACGATATTCGAA CACCTCTTATGATACGATGTCGAAGTTTATAAC	213	MW288972
		Emult61	TTTTTCGCGCATTTATTGCTTTTCAGAATTCAATATTCCAAATCATTCAAATTCCATGAC ACATATTTTTTCCTACATCATCAGTCCAACATTTTCACTGATATTTTTATTATTGTCGTT ACCATAACTTTCAGAATGTACTAAATAGACGTTCATGCATGCATGTAGAACGATATTCGA ACTCCTCTTATGATAAGATGTCGAAGTTTATAAC	214	MW288973

E. pungens	‘Maculata’	Epung64	TTTTTGCCGCATTTATTGACTTCAGAATTCGTTATTCCAAATCATTCAAATTTCATGAAA<br>TATATTTTTTGCTAAATCTTCTTTGCAACTTTTCTACTTATACTTTTATTTTTATCGTTA<br>CCATAAGTTTAAGAATGTACTAAATAGACGTTCATGCATGCATGTAGAACGATATTCGAA<br>CTCTTCTTATGATTTGGTTTCGAAGTTTATAAC	213	MW288974
		Epung65	CTTTTGCCGCATTTATTGATCTAAGGATTCGCTATTCCAAATCATTCAAATTTCATGAAA<br>CATATTTTTTGCTAAATCATCTTTCCAACTTTTCTACTTATACTTTTATTTTTGTCGTTA<br>CCATAAGTTTAAGAATGTACTAAATAGACGTTCATGCATGCATGTAGAACGATATTCGAA<br>CTCTTCTTATGATTTGGTTTCGATGTTTATAAC	213	MW288975
		Epung72	CTTTTGCCGCATTTATTGATCTCAGAATTCGATATTCCAAATCATTCAAATTTCATGAAA<br>CATATTTTTTGCTAAATCATGTTTCCAACTTTTCTACTTACACTTTTATTTTTATCGTTA<br>CCATAAGTTTAAGAATGTACTAAATAGACGTTCATGCATGCATGTAGAACGATATTCGAA<br>CTCTTCTTATGATTTGGTTTCGAAGTTTATAAC	213	MW288976
E. umbellata	Sweet’n’Sour	EumbSS75	TTTTTGGCGCATTTATTGCTTTCAGAATTCAATATTCCAAATCATTCAAATTCCAAGACA<br>CATATTTTTTGCTACATCATCAGTCCAACATTTTCACTGATATTTTTATTATTGTCGTTA<br>CCATAACTTTCAGAATGTACTAAATAGACGTTCACGCATGCATGTAGAACGATATTCGAA<br>CTCCTCTTATGATAAGATGTCGAAGTTTATAAC	213	MW288989
	Fortunella	EumbF79	TTTTTGGCGCATTTATTGCTTTCAGAATTCAATATTCCAAATCATTCAAATTCCAAGACA<br>CATATTTTTTGCTACATCATCAGTCCAACATTTTCACAGATATTTTAATTATTGTCGTTA<br>CCATAACTTTCAGAATATACTAAATAGACGTTCACACATGCATGTAGAACGATATTCGAA<br>CTCCTCTTATGATAAGATGTCGAAGTTTATAAC	213	MW288977
		EumbF82	TTTTTGGTGCATTTATTGCTTTCAGAATTCAATATTCCAAATCATTCAAATTCCCTGACA<br>CATATTTTTTGCTACATCATCAGTCCAACATTTTCACTGATATTTTTATTATTGTCGTTA<br>CCATAACTTTCATAATGTACTAAATAGACGTTCACGCATGCATGTAGAACGATATTCGAA<br>CACCTCTTATGATAAGATGTCGAAGTTTATAAC	213	MW288978
		EumbF84	TTTTTGGTGCATTTATTGCTTTCAGAATTCAATATTCCAAATCATTCAAATTCCCTGACA<br>CATATTTTTTGCTACATCATCAGTCCAACATTTTCACTGATATTTTTATTATTGTCGTTA<br>CCATAACTTTCATAATGTACTAAATAGACGTTCACGCATGCATGTAGAACGATATTCGAA<br>CACCTCTTATGATAAGATGTCGAAGTTTATAAC	213	MW288979
		EumbF85	TTTTTGGCGCATTTATTGCTTTTAGAATTCAATATTCCAAATCATTCAAATTCCAACACA<br>CATATTTTTTGCTACATCATCAGTCCAACATTTTCACTGATATTTTTATTATTGTCGTTA<br>CCATAACTTTCAGAATGTACTAAATAGACGTTTACGCATGCATGTAGAACGATATTCGAA<br>CACCTCTTATGATAGGATGTCGAAGTTTATAAC	213	MW288980
		EumbF86	TTTTTGGTGCATTTATTGCTTTCAGAATTCAATAGTCCAAATCATTCAAATTCCAAAACA<br>CATATTTTTTGCTACATCATCAGTCCAACATTTTCTCTGATATTTTTATTATTGTCGTTA	213	MW288981

			CCATAACTTTCAGAATGTACTAAATAGACGTTCACGCATGCATGTAGAACGATATTCGAA CTCCTCTTATGATAAGATGTCGAAGTTTATAAC		
	Brilliant Rose	EumbBR88	TTTTTGGCGCATTTATTGCTTTCAGAATTCAATATTCCAAATCATACAAATTCCATGACA CATATTTTTTGCTACATCACCAGTCCAACATTTTCACTGATATTTTTATTATTGTCGTTA CCATAACTTTCAGAATGTACTAAATAGACGTTCACGCATGCATGTATAACGATATTCGAA CTCCTCTTATGATAAGATGTCGAAGTTTATAAC	213	MW288982
		EumbBR89	ATTTTTGGCGCATTTATAGCTTTCAGAATTCAATATTCCAAATCATTCAAATTCCAAGAC CCATATTTTTTGCTACATCATCAGTCCAACATTTTCACTGATATTTTTATTATTGTCGTT ACCATAACTTTCAGAATGTACTAAATAAACGTTCACGCATGCATGTAGAACGATATTCGA ACTCTTCTTATGATAAGATGTCGAAGTTTATAAC	214	MW288983
		EumbBR91	TTTTTGGCGCATTTATTGCTTTCGGAATTCAATATTCCAAATCATTCAAATTCCATGACA CATATTTTTTGCTACATCATCAGTCCAACATTTTCACTGATATTTTTATTATTGTCGTTA CCATAACTTTGAGAATGTACTAAATAGACGTTCACGCATGCATGTAGAACGATATTCGAA CACCTCTTATGATACGATGTCGAAGTTTATAAC	213	MW288984
		EumbBR92	TTTTTGGCGCATTTATTGCTTTCAGAATTCAATATTCCAAATCATTCAAATTCCAAGACA CATAATTTTTACTACATCATCAGTCCAACATTTTCACTGATATTTTTATTATTGTCGTTA CCATAACTTTCAGAATGTACTAAATAGACGTTCACGCATGCATGTAGAACGATATTCGAA CTCTTCTTATGATAAGATGTCGAAGTTTATAAC	213	MW288985
		EumbBR98	TTTTTGGCACATTTATTGCTTTCAGAATTCAATATTCCAAATCATTCAAATTCCAAGACA CATATTTTTTGCTACATCTTTAGTCCAACATTTTCACTGATATTTTTGTTATGTTCGTTA CCATAACTTTCAGAATGTACTAAATAGACGTTCACGAATGCATGTAGAACGATATTCGAA CTCCTCTTATGATAAGATGTCGAAGTTTATAAC	213	MW288986
		EumbBR100	TTTTTGGCGCATTTATTGCTTTTAGAATTCAATATTCCAAATCATTCAAATTCCCTGACA CATATTTTTTGCTACATCATCAGTCCAACATTTTCACTGATATTTTTATTATTGTCGTTA CCATAACTTTCAGAATGTACTAAATAGACGTTCACGCATGCATGTAGAACGATATTCGAA CTCTTCTTATGATAAGATGTCGAAGTTTATAAC	213	MW288987
		EumbBR101	ATTTTTGGCGCATTTATTGCTTTCAGAATTCAATATTCCGAATCATTCAAATTCCAAGAC ACATATTTTTTGCTACATCATCAGTCCAACATTTTCACTGATATTTTTATTATTGTCGTT ACCATAACTTTCAGAATGTACTAAATAGACGTTCACGCATGCATGTGGAACGATATTCGA ACTCCTCTTATGATAAGATGTCGAAGTTTACAAC	214	MW288988
	without variety	EumbB104	TTTTTGGCACATTTATTGCTCTTAGAATTCAATATTCCAAATCATTCAAATTCCATGACA CATATTTTTTGCTACATCACCCGTCCAACATTTTCACTGATATTTTTATTATTGTCATTA CCATAACTTTCAAAATGTACTAAATAGACGTTCACGCATGCATGTAGAACGATATTCGAA CTCCTCTTATGATAAGATGTCGAACTTTGTAAC	213	MW288990

		EumbB109	TTTTTGGCGAATTTATTGCTTTCAGAATTCAATATTCCAAATCATTCAAATTCTCTGACA<br>CATATTTTTTGCTACATCATCAGTCCAACATTTTCACTGATATTTTTATTATTGTCGTTA<br>CCATAACTTTCAGAATGTACTAAATAGACGTTCACGCATGCATGTATAACGATATTGGAA<br>CTCCTCTTATGATAAGATGTCGAAGTTTATAAC	213	MW288991
		EumbB110	TTTTTGGCGCATTTATTGCTTTCAGAATTCAATATTCCAAATCATTCAAATTCTATGACA<br>CATATTTTTTGATACATCATCAGTCCAACATTTTCACTGATATTTTTATTATTGTCGTTA<br>CCATAACTTTCAGAATGTACTAAATAGACGTTCACGCATGCATGTAGAACGATATTCGAA<br>CTCCTCTTATGATAAGATGTCGAAGTTTATAAC	213	MW288992
		EumbB111	TCTTTGGCTCATTTATTGCTTTCAGAATTCAATATTCCAAATCATTCAAATTTCATGACA<br>CATATTTTTTGCTACATCATCAGTACAACATTTTCACTGATATTTTTATTATTGTCATTA<br>CCATAACTTTCAGAATGTACTAAATAGACGTTCACGCATGCATGTAGAACGATATTCGAA<br>CTCCTCTTATGATAAGATGTCGAAGTTTATAGC	213	MW288993
		EumbB112	TTTTTGGCGCATTTATTGCTTTCACAATTCAATATTCCAAATCATTCAAATTCCCTGACA<br>CATATTTTTTGCTACATCATCAGTCCAACATTTTCACTGATATTTTTATTATTGTCGTTA<br>CCATAACTTTTAGAATGTACTAAATAGACGTTCACGCATGGATGTAGAACGATATTCGAA<br>CTCCTCTTATGATAAGATGTCGAAGTTTATAAC	213	MW288994
		EumbB113	TTTTTGGCGCATTTATTGCTTTCAGAATTCAATATTCCAAATCATTCAAATTCTATGACA<br>CATATTTTTTGCTACATCATCAGTCCAACATTTTCACTGATATTTTTATTATTGTCGTTA<br>CCATAACTTTCAGAATGTCCTAAATAGACGTTCACGCATGCATGTAGAACGATATTCGAA<br>CTCCTCTTATGATATGATGTCGAAGTTTATAAC	213	MW288995
		EumbB115	TCTTTGGCTCATTTATTGCTTTCAGAATTCAATATTCCAAATCATTCAAATTTCATGACA<br>CATATTTTTTGCTACATCATCAGTACAACATTTTCACTGATATTTTTATTATTGTCATTA<br>CCATAACTTTCAGAATGTACTAAATAGACGTTCACGCATGCATGTAGAACGATATTCGAA<br>CTCCTCTTATGATAAGATGTCGAAGTTTATAGC	213	MW288996
		EumbB116	TTTTTGGCGCATTTATTGCTTTCAGAATTCAACATTCCAAATCATTCAAATTCCATGACA<br>CATATTTTTTGCTACATCATCAGTCCAACATTTTCACTGATATTTTTATTATTGTCGTTA<br>CAATAACTTTCAGAATGTATTAAATAGACGTTCACGCATGCATGTAGAACGATATTCGAA<br>CTCCTCTTATGATAAGATGTCGAAGTTTGCAAC	213	MW288997
		EumbB117	TCTTTGGCTCATTTATTGCTTTCAGAATTCAATATTCCAAATCATTCAAATTTCATGACA<br>CATATTTTTTGCTACATCATCAGTACAACATTTTCACTGATATTTTTATTATTGTCATTA<br>CCATAACTTTCAGAATGTACTAAATAGACGTTCACGCATGCATGTAGAACGATATTCGAA<br>CTCCTCTTATGATAAGATGTCGAAGTTTATAGC	213	MW288998
		EumbB118	TTTTTGGCGAATTTATTGCTTTCAGAATTCAATATTCCAAATCATTCAAATTCTCTGACA<br>CATATTTTTTGCTACATCATCAGTCCAACATTTTCACTGATATTTTTATTATTGTCGTTA	213	MW288999

			CCATAACTTTCAGAATGTACTAAATAGACGTTCACGCATGCATGTATAACGATATTGGAA CTCCTCTTATGATAAGATGTCGAAGTTTATAAC		
		EumbB119	TTTTTGGCGAATTTATTGCTTTCAGAATTCAATATTCCAAATCATTCAAATTCTCTGACA CATATTTTTTGCTACATCATCAGTCCAACATTTTCACTGATATTTTTATTATTGTCGTTA CCATAACTTTCAGAATGTACTAAATAGACGTTCACGCATGCATGTATAACGATATTGGAA CTCCTCTTATGATAAGATGTCGAAGTTTATAAC	213	MW289000
		EumbB120	TTTTTGGCGAATTTATTGCTTTCAGAATTCAATATTCCAAATCATTCAAATTCTCTGACA CATATTTTTTGCTACATCATCAGTCCAACATTTTCACTGATATTTTTATTATTGTCGTTA CCATAACTTTCAGAATGTACTAAATAGACGTTCACGCATGCATGTATAACGATATTGGAA CTCCTCTTATGATAAGATGTCGAAGTTTATAAC	213	MW289001
		EumbB121	TTTTTGGCGCATTTATTGCTTTCACAATTCAATATTCCAAATCATTCAAATTCCCTGACA CATATTTTTTGCTACATCATCAGTCCAACATTTTCACTGATATTTTTATTATTGTCGTTA CCATAACTTTTAGAATGTACTAAATAGACGTTCACGCATGGATGTAGAACGATATTCGAA CTCCTCTTATGATAAGATGTCGAAGTTTATAAC	213	MW289002
		EumbB124	TTTTTGGCGCATTTATTGCTTTCAGAATTCAATATTCCAAATCATTCAAATTCCAAGACA CATATTTTTTGCTACATCATCAGTCCAACATTTTCACTGATATTTTTATTATTGTCGTTA CCATAACTTTCAGAATGTACTAAATAGACGTTCACGCATGCATGTATAACGATATTCGAA CTCCTCTTATGATAAGATGTCGAAGTTTATAAC	213	MW289003
		EumbB125	TTTTTGGTGCATTTATTGCTTTCACAATTCAATATTCCAAATCATTCAAATTCCCTGACA CATATTTTTTGCTACATCTCCAGTCCAACATTTTCACTGATATTTTTATTATTGTCGTTT CCATAACTTTCAGAATGTACTAAATAGACGTTCACGCATGCATGTAGAACGATATTCGAA CACCTCTTATGATAAGATGTCGAAGTTTATAAC	213	MW289004
		EumbB126	TTTTTGGCGCATTTATTGCTTTCAGAATTCAATATTCCAAATCATTCAAATTCTATGACA CATATTTTTTGATACATCATCAGTCCAACATTTTCACTGATATTTTTATTATTGTCGTTA CCATAACTTTCAGAATGTACTAAATAGACGTTCACGCATGCATGTAGAACGATATTCGAA CTCCTCTTATGATAAGATGTCGAAGTTTATAAC	213	MW289005
Sh. argentea		Sharg5	CACTTCCTTCCCTTTTTTTTTTTTTTTTCAGAAAACGAATACGTTAATATATGTTACAATC AATAAATAGAGTTTACATTGCTTATTCCTTTCTTATACATAAACGTTGAAATCTATAAT	119	MW288952
		Sharg8	CTTTTCCCCCCCTTTTTTTTTTTTGTTTTTTTTCAGAAAACGAATACGTTAATATATGTT ACAATCAATAAATAGAGTTTACATTGCTTATTCCTTTCTTATACATAAACGTTGAAATCT ATAAT	125	MW288953
		Sharg9	CTCTTCCCCCACTTTTTTTTTTTTTTCAGAAAACGAATACGTTAATATATGTTACAATCAA TAAATAGAGTTTACATTGCTTATTCCTTTCTTATACATAAACGTGGAAATCTATAAT	117	MW288954
		Sharg10	CTCTTCCCCCACTTTTTTTTTTTTTTTTCAGAAAACGAATACGTTAATATATGTTACAATC	119	MW288955

			AATAAATAGAGTTTACATTGCTTATTCCTTTCTTATACATAAACGTTGAAATCTATAAT		
		Sharg14	CCTTTTCCCCCCCCCTTTTTTTTTTTTTTTTTTTTTTTTTTTTTTTTTTTTTCAGAAAAC GAATACGTTAATATATGTTACAATCAATAAATAGAGTTTACATTGCTTATTCCTTTCTTA TACATAAACGTTGAAATCTATAAT	144	MW288956
Sh. canadensis		Shcan25	TTTTTCCATCTCATTACATTTTTTTTTTCCGGTACGAAATGCCTTATTCTTGTTTATAAA AATAATGTTAATACTTCTTAACATTAATTTGAAAAATTAAAGACCGCAAAAAAACAATTT ATAAGATCTCTTCTTATATGTAATAAGTGGTTCATCTGAC	160	MW288957
