# Supplementary material for: A Comparative Study of 5S rDNA Non-Transcribed Spacers in Elaeagnaceae Species
Source: Plants (Basel). 2020 Dec 23;10(1):4. doi: 10.3390/plants10010004 (PMC7822202; doi:10.3390/plants10010004)
Supplement: Supplementary file 1 [file plants-10-00004-s001.zip › Table S3.pdf]

Table S3. Mutations in the *Elaeagnus umbellata* NTSs

Variety	Mutation	Positions	Number of transitions	Number of tranversions	Total
Fortnella	C/T	8, 23, 153	6		14
	A/G	137, 156, 195			
	T/G	35, 133		8	
	A/C	55			
	A/T	56, 96, 98, 107, 182			
	C/G				
Brilliant Rose	C/T	24, 81, 82, 185, 211	12		26
	A/G	10, 25, 40, 72, 109, 148, 167			
	T/G	114, 115, 168		14	
	A/C	56, 61, 158, 196			
	A/T	18, 47, 57, 66, 80, 183			
	C/G	132			
without variety	C/T	2, 8, 21, 23, 33, 53, 54, 80, 131, 140, 210	15		32
	A/G	117, 133, 209, 212			
	T/G	167		17	
	A/C	10, 55, 72, 82, 85, 122, 139			
	A/T	56, 79, 120, 182, 195			
	C/G	25, 161, 177, 205			
