# Supplementary material for: A Comparative Study of 5S rDNA Non-Transcribed Spacers in Elaeagnaceae Species
Source: Plants (Basel). 2020 Dec 23;10(1):4. doi: 10.3390/plants10010004 (PMC7822202; doi:10.3390/plants10010004)
Supplement: Supplementary file 1 [file plants-10-00004-s001.zip › Table S4.pdf]

Table S4. Mutations in the *Elaeagnus* spp. NTSs

Mutation	Number of mutations	Number of transitions	Number of tranversions	Total
C/T	43	63		129
A/G	20			
T/G	8		66	
A/C	21			
A/T	28			
C/G	9			
