# Supplementary material for: A Comparative Study of 5S rDNA Non-Transcribed Spacers in Elaeagnaceae Species
Source: Plants (Basel). 2020 Dec 23;10(1):4. doi: 10.3390/plants10010004 (PMC7822202; doi:10.3390/plants10010004)
Supplement: Supplementary file 1 [file plants-10-00004-s001.zip › Table S6.pdf]

Table S6. A comparative analysis of the average levels of interspecies (intervariety for *Elaeagnus umbellata*) identity by *t*-tests.

	Elaeagnus angustifolia	*Elaeagnus commutata*	*Elaeagnus multiflora*	*Elaeagnus pungens*	*Elaeagnus umbellata* Fortunella	*Elaeagnus umbellata* Brilliant Rose	*Elaeagnus umbellata* B	*Elaeagnus umbellata* Sweet'n'Sour
Elaeagnus angustifolia	96,07±0,47	60,83±0,25	57,03±0,26	58,67±0,20	56,87±0,18	56,81±0,19	55,57±0,11	55,17±0,18
Elaeagnus commutata	60,83±0,25	96,30±0,59	60,56±0,15	60,93±0,19	62,12±0,18	61,29±0,10	61,13±0,07	61,60±0,27
Elaeagnus multiflora	57,03±0,26	60,56±0,15	95,70±0,59	84,50±0,18	95,56±0,26	95,54±0,20	95,53±0,12	97,20±055
Elaeagnus pungens	58,67±0,20	60,93±0,19	84,50±0,18	95,00±0,71	84,00±0,00	84,95±0,18	84,58±0,11	86,00±0,00
Elaeagnus umbellata Fortunella	56,87±0,18	62,12±0,18	95,56±0,26	84,00±0,00	96,10±0,48	95,51±0,15	95,31±0,12	97,40±0,27
Elaeagnus umbellata Brilliant Rose	56,81±0,19	61,29±0,10	95,54±0,20	84,95±0,18	95,51±0,15	95,48±0,23	95,23±0,13	97,43±0,32
Elaeagnus umbellata B	55,57±0,11	61,13±0,07	95,53±0,12	84,58±0,11	95,31±0,12	95,23±0,13	95,78±0,17	96,94±0,96

- the average value of intraspecies identity, which is compared with correspond values in line

- the differences are statistically significant

- the differences are not statistically significant

- the empirical values of the t-criteria are in the zone of uncertainty

The empirical values of t-criteria

	Elaeagnus angustifolia	*Elaeagnus commutata*	*Elaeagnus multiflora*	*Elaeagnus pungens*	*Elaeagnus umbellata* Fortunella	*Elaeagnus umbellata* Brilliant Rose	*Elaeagnus umbellata* B	*Elaeagnus umbellata* Sweet'n'Sour
Elaeagnus angustifolia		74,2	78,7	80,4	97,1	95,2	121	55,8
Elaeagnus commutata	66		86,5	70,2	76,8	99,7	131,3	42,2
Elaeagnus multiflora	59,5	85		21,8	0,3	0,3	0,4	1,7
Elaeagnus pungens	67,9	72,1	22,3		49,2	19,8	22,5	15,8
Elaeagnus umbellata Fortunella	96,8	85,1	1,1	32,7		1,6	2	2,2
Elaeagnus umbellata Brilliant Rose	125	155,2	0,2	37,6	0,1		0,8	4,6
Elaeagnus umbellata B	185,9	160,8	1,1	40	2	2,5		2,4

The critical values of t-criteria

	Elaeagnus angustifolia	*Elaeagnus commutata*	*Elaeagnus multiflora*	*Elaeagnus pungens*	*Elaeagnus umbellata* Fortunella	*Elaeagnus umbellata* Brilliant Rose	*Elaeagnus umbellata* B	*Elaeagnus umbellata* Sweet'n'Sour
Elaeagnus angustifolia		2,02	2,02	2,04	2,02	2	1,97	2,09
Elaeagnus commutata	2,02		2,04	2,07	2,04	2,02	1,99	2,16
Elaeagnus multiflora	2,08	2,04		2,07	2,04	2,02	1,99	2,16
Elaeagnus pungens	2,09	2,12	2,12		2,12	2,07	2	2,78
Elaeagnus umbellata Fortunella	2,02	2,04	2,04	2,07		2,02	1,99/2,63*	2,13/2,95*
Elaeagnus umbellata Brilliant Rose	1,99	2	2	2,02	2		1,97	2,06
Elaeagnus umbellata B	1,96	1,97	1,97	1,97	1,97/2,61	1,96/2,58*		1,97/2,61

* $p \leq 0.05$/$p \leq 0.01$
